# In Vivo Genotoxicity Evaluation of a Stilbene Extract Prior to Its Use as a Natural Additive: A Combination of the Micronucleus Test and the Comet Assay

**DOI:** 10.3390/foods10020439

**Published:** 2021-02-17

**Authors:** Concepción Medrano-Padial, María Puerto, Ana Isabel Prieto, Nahúm Ayala, Pauline Beaumont, Caroline Rouger, Stéphanie Krisa, Silvia Pichardo

**Affiliations:** 1 Area of Toxicology, Faculty of Pharmacy, Universidad de Sevilla, C/Profesor García González n°2, 41012 Seville, Spain; cmpadial@us.es (C.M.-P.); anaprieto@us.es (A.I.P.); spichardo@us.es (S.P.); 2 Veterinary Faculty, Universidad de Córdoba, Campus de Rabanales, Edificio de Sanidad Animal, 14071 Córdoba, Spain; nahum.ayala@uco.es; 3 Unité de Recherche Œnologie, Molécules d’Intérêt Biologique, EA4577, USC 1366 INRAE, Université de Bordeaux, Institut des Sciences de la Vigne et du Vin, 210 Chemin de Leysotte, 33882 Villenave d’Ornon, France; pauline.beaumont@u-bordeaux.fr (P.B.); caroline.rouger@u-bordeaux.fr (C.R.); stephanie.krisa@u-bordeaux.fr (S.K.)

**Keywords:** stilbenes, genotoxicity, comet assay, micronucleus, in vivo, rat

## Abstract

Genotoxic data of substances that could be used as food additives are required by the European Food Safety Authority. In this sense, the use of an extract from grapevine shoots containing a stilbene richness of 99% (ST-99), due to its antioxidant and antibacterial activities, has been proposed as an alternative to sulfur dioxide in wine. The aim of this work was to study, for the first time, the in vivo genotoxic effects produced in rats orally exposed to 90, 180, or 360 mg ST-99/kg body weight at 0, 24, and 45 h. The combination of micronucleus assay in bone marrow (OECD 474) and standard (OECD 489) and enzyme-modified comet assay was used to determine the genotoxicity on cells isolated from stomach, liver, and blood of exposed animals. The ST-99 revealed no in vivo genotoxicity. These results were corroborated by analytical studies that confirm the presence of stilbenes and their metabolites in plasma and tissues. Moreover, to complete these findings, a histopathological study was performed under light microscopy in liver and stomach showing only slight modifications in both organs at the highest concentration used. The present work confirms that this extract is not genotoxic presenting a good profile for its potential application as a preservative in the wine industry.

## 1. Introduction

Many natural compounds such stilbenes or their analogues have awakened the interest of the scientific community because of their potential use as additives in the food industry [1,2]. Although SO_2_ is usually added to wine for its antioxidant and antimicrobial properties, it has been clearly shown that there is risk to human health such as bronchospasm, bradycardia, gastrointestinal symptoms, urticaria, angioedema, hypotension, shock, and even anaphylactic reactions in sensitive individuals [3,4]. Today’s consumers demand high quality foods that are free from chemical substances, fresh tasting, microbiologically safe, and with an extended shelf-life [5]. In this regard, favorable results have been obtained with the use of natural compounds such as stilbenes and flavonoids. Some studies have demonstrated that phenolic compounds naturally found in grape extracts present a high antimicrobial activity against bacteria that cause numerous deterioration in wine [6,7,8]. Moreover, these compounds exhibited great antioxidant capacity such as scavenging of free radicals [9,10,11,12]. Thus, wines treated with grapevine extracts presented excellent enological parameters and higher color intensity than wines with SO_2_ treatment [5,13,14]. Considering this background, preliminary studies have determined the possibility of using a grapevine shoot extract (ST-99 extract) as a natural additive to replace SO_2_ in wines [4,5,12,13,14]. The ST-99 extract was obtained from grapevine shoots harvested in Bordeaux region (France). It contains at least 99% of stilbenes (w/w), of which main stilbenes are trans-ε-viniferin (70%) and trans-resveratrol (18%). Other stilbenes found in a lower percentage are vitisin B (4%), w-viniferin (4%), cis- ε-viniferin (1%), miyabenol C (1.5%), and cis-resveratrol (0.5%). In preliminary studies, this extract showed good antimicrobial activity, and it did not affect the sensory properties nor quality of wines [15]. Moreover, it exhibited high antioxidant activity in human cell lines [16]. 

In a first toxicity evaluation approach, in vitro cytotoxicity studies were performed in our laboratory, demonstrating that ST-99 induced damage in human intestinal and hepatic cell lines and caused morphological changes from 30–40 µg/mL [17]. Moreover, in order to test the genotoxic effects of substances in food and feed, the European Food Safety Authority (EFSA) recommends a stepwise approach, beginning with the Ames test and in vitro micronucleus assay (MN) [18]. In the case of ST-99 extract, no mutagenic potential was found at concentrations from 48 to 5000 µg/plate in any of the five strains of *Salmonella typhimurium* used either in presence or absence of the microsomal fraction S9 (S9 mix). The MN assay in L5178Y TK ^+/−^cells indicated that, in the absence of S9 mix, none of the concentrations tested (4–64 µg/mL) increased the frequency of binucleated cells with MN (BNMN). However, a significant increase in BNMN was observed with S9 mix after the exposure of the highest concentration studied (60 µg/mL). Moreover, the standard and modified comet assay was also performed, showing negative results in Caco-2 and HepG2 cell lines [19]. In addition to the in vitro assays, in order to use ST-99 in the food industry, it is necessary to check its safety in at least one in vivo study [20]. Three different in vivo assays are recommended by EFSA to evaluate the genotoxicity of substances: mammalian erythrocyte micronucleus test (Organisation for Economic Co-operation and Development (OECD) TG 474), transgenic rodent somatic and germ cell gene mutation assays (OECD TG 488), and in vivo Comet assay [18,20]. In this sense, the modified comet assay for detection of oxidative DNA lesions can be recommended since most DNA damaging agents induce other lesions such as oxidized and alkylated bases [21].

To our knowledge, the data published related to the in vivo genotoxicity of stilbene-rich extracts containing ε-viniferin and/or *trans*-resveratrol are very scarce, and none of the published works have followed the in vivo tests required by EFSA. Only Tatefuji et al. [22] evaluated an extract from the seeds of melinjo (*Gnetum gnemon L*.) rich in dimers of resveratrol, *trans*-resveratrol and their glycosides using the MN test in Wistar rats administered by gavage for 2 days. The results indicated that this extract (up to 4000 mg/kg of body weight (b.w.)/day) did not possess genotoxic potential and it has been used as food ingredients for a long time.

On the other hand, no in vivo studies about the safety profile of ε-viniferin (the main compound of ST-99 extract) were described in the scientific literature. However, with respect to *trans*-resveratrol, which is the 18% of the extract, the EFSA the Panel on Dietetic Products, Nutrition, and Allergies considered that this stilbene is not genotoxic. In this sense, although *trans*-resveratrol alone has exhibited no genotoxic effects in vivo, the risk assessment of extracts or mixtures that contain chemically related molecules or mixtures of isomers is necessary since a potentiation of the genotoxic and mutagenic effect could be found [23,24].

Considering all these facts, this work is focused on assessing the in vivo genotoxicity of ST-99 extract by performing a combination of the MN test in bone marrow and the comet assay on cells isolated from stomach and liver of rats proposed by Bowen et al. [19], following OECD 474 and OECD 489 guidelines [25,26]. This combined approach has certain advantages over performing these assays individually. It is more sensitive and specific since MN test determinates the structural and numerical chromosomal damage and the comet assay detects DNA damage. Furthermore, it was performed in a limited number of animals complying with the 3Rs principles (Replace, Reduce, and Refine) [27]. Moreover, our group has previous experience performing the MN as well as comet assay simultaneously to detect the genotoxic effects of different substances in rats [28,29,30]. This evaluation was completed with a detailed analytical study to ensure that ST-99 extract effectively reached the studied tissue. Finally, histopathological examination was performed in order to provide useful information for an accurate risk assessment. 

## 2. Materials and Methods

### 2.1. Supplies and Chemicals 

Chemicals were provided by Sigma-Aldrich (Madrid, Spain), C-viral S.L (Seville, Spain), Gibco (Biomol, Seville, Spain), Moltox (Trinova, Biochem, Germany), and VWR International Eurolab (Barcelona, Spain). 

ε-Viniferin and trans-resveratrol used for calibration curves were extracted and purified from grape shoot extract as previously described using chromatographic techniques. Methanol (MeOH) was purchased from VWR Chemicals and formic acid and high-purity grade UPLC-MS acetonitrile were purchased from Fisher Chemicals.

### 2.2. Animal Hosting and Nourishing Conditions

Nine-week old Wistar rats, strain RjHan: WI (type outbred rats) were provided by Charles Rivers (Iffa Credo, Saint Germain sur l’Arbresle, France). Animals were humanly cared for by the protection of animals utilized for scientific purposes following the Directive 2010/63/UE. Moreover, the Ethics Committee on Animal Experimentation of the University of Sevilla authorized the in vivo experiment. 

All animals were weighed and accommodated into polycarbonate cages with stainless steel covers upon arrival. Then, they were acclimatized to environmental conditions (12-h dark/light cycle, controlled temperature (23 ± 1 °C), relative humidity (55 ± 10%) for 1 week before the experiment. During this time, the animals were fed with standard laboratory diet Harlan, 2014; Harlan Laboratories, Barcelona, Spain and water ad libitum.

### 2.3. Stilbene Enriched Extract

Grapevine shoots were harvested in 2015 in Bordeaux region (France) and were composed of a mixture of Merlot and Cabernet Sauvignon varieties of *Vitis vinifera*. ST-99 extract was obtained from grapevine shoots as described in Gutiérrez-Escobar et al. [15]. Grapevine shoots of *Vitis vinifera* cv. (1 kg) were extracted with a mix of acetone–water (6:4, *v/v*) at room temperature under agitation, twice for 12 h. After filtration, the solution was evaporated and lyophilized. The extract was deposited on an Amberlite XAD-7 column and washed with water, and eluted with acetone.

The extract contained 99% of total stilbenes (*w/w*), being found *trans*-ε-viniferin (70%) and *trans*-resveratrol (18%) the main stilbenes. Other stilbenes found in a lower percentage are vitisin B (4%), w-viniferin (4%), cis- ε-viniferin (1%), miyabenol C (1.5%), and cis-resveratrol (0.5%). 

### 2.4. Experimental Design and Treatment

In order to calculate the experimental doses, it has been taken in consideration the estimated dose of ST-99 extract that will reach to consumer and an uncertainty factor (UF) of 100 (factor 10 for inter-species variability and 10 to cover inter-individual human variability) introduced by Lehman and Fitzhugh [31] for extrapolating from animal toxicity data to safe levels of human exposure to food additives and pesticide residues, and later it was adopted by the Joint FAO/WHO Expert Committee on Food Additives (JECFA). Thus, the three tested doses were 360 mg ST-99/kg b.w., 180 mg ST-99/kg b.w., and 90 mg ST-99/kg b.w All doses were prepared in a final volume of 1 mL (0.1% DMSO).

Following OECD guidelines 474 and 489 [25,26], 5 animals per group and 3 animals for positive controls were used per sex. After acclimation, 28 male and 28 female rats were weighted in order to ensure that weight variation did not exceed ± 20% and were randomly divided into 5 groups: 1 negative control group (5 male and 5 female rats) treated with water; 1 solvent control group (0.1% DMSO, vehicle) (5 male and 5 female rats), 1 positive control group (3 male and 3 female rats) exposed to 200 mg/kg b.w. ethylmethanesulfonate (EMS), and 3 exposed groups (5 male and 5 female rats per group) treated with 90, 180, or 360 mg ST-99/kg b.w.

According to Bowen et al. (2011) [24], animals were dosed by gavage using an enteral feeding tube (Vygon, Ecouen, France) at 0 h, 24 h and 45 h and sacrificed 3 h after the final dose administration for combined comet and MN endpoints. Body weight and clinical signs were recorded during treatment. 

### 2.5. Sample Collection

Blood samples (3–5 mL) were collected in Vacutainer^®^ sodium heparin tubes (Becton Dickinson, Rutherford, NJ, USA). Liver and stomach were removed, dissected, rinsed with cold saline solution, and weighed. Then, stomach and liver (0.5 g approximately) and blood samples were quickly processed for the comet assay as is described in Section 2.6. 

MN samples were collected from the bone marrow of both femurs of each animal and immediately processed. 

### 2.6. MN Assay

The recommendations of OECD guideline 474 [25] and Corcuera et al. [32] were followed to perform MN assay. The bone marrow cells were suspended in a drop of fetal bovine serum. Two slides, 1 per femur of each animal were prepared. Then, they were fixed in absolute methanol, air dried, and stained with 10% Giemsa. 

The polychromatic erythrocytes (PCE) among total erythrocytes (normochromatic erythrocytes (NCE) + (PCE)) ratio and the PCE among NCE ratio were calculated by counting 500 erythrocytes per animal. 

The incidence of micronucleated immature erythrocytes (MN-PCEs) was calculated by counting a total of 5000 PCE per animal and results were expressed as % MN. 

### 2.7. Isolation of Single-Cell Suspensions for the Comet Assay

Following Corcuera et al. [32] and Mellado-García et al. [28], single cell suspensions from both tissues were isolated. Liver and stomach were washed with Merchant’s buffer (MB) (0.14 M NaCl, 1.47 mM KH_2_PO_4_, 2.7 mM KCl, 8.1 mM Na_2_HPO_4_, 10 mM Na_2_EDTA, pH 7.4), and a portion of each were homogenized in cold. The homogenates were centrifuged, filtered, and mixed with 5 mL buffer until slide preparation. 

Heparinized blood samples were mixed *v/v* (1/1) with phosphate buffered saline (PBS) solution and the lymphocytes were isolated with Histopaque® (SigmaAldrich, Madrid, Spain) and centrifuged (400 *g*, 30 min) [33]. Finally, the cells were washed twice with PBS and re-suspended at a concentration of 2 × 10^5^ cells/mL.

### 2.8. Standard and Enzyme-Modified Comet Assay

The recommendations of OECD guideline 489 [26] were followed to perform the standard comet assay. Cells suspension was mixed with 0.5% low-melting point agarose for blood samples [33], while for stomach and liver cells were mixed with 1% low-melting point agarose and both samples were placed on a microscope slide [28,33]. Then, the standard and modified comet assays were performed as previously described by Mellado-García et al. [28]. Briefly, slides were in lysis at 4 °C during at least 1 h and then washed 3 times for 5 min with enzyme buffer (40 mM HEPES; 0.1M KCl; 0.5 mM EDTA; 0.2 mg/mL bovine serum albumin; pH 8). Afterwards, 2 gels in each slide were exposed to 30 μL of lysis solution, enzyme buffer alone (buffer F), buffer F containing Formamidopyrimidine DNA glycosylase (FPG), or Endonuclease III (Endo III) during 30 min in a metal box at 37 °C. After exposure time, electrophoresis was performed for 20 min, 0.81 V/cm up to 400 mA and DNA was neutralized in PBS, washed with water, and fixed with 70% and absolute ethanol before staining. 

Using image analysis software Comet Assay IV (Perceptive Instruments, Suffolk, UK), 150 randomly selected nuclei per animal were analyzed. 

The % DNA in tail represents DNA strand breaks and oxidized damage in DNA bases. Endo III and FPG sensitive sites were calculated by subtracting the % of DNA in tail after enzyme buffer incubation from the % of DNA in tail after repair enzymes incubation. 

### 2.9. Determination of Stilbenes in Plasma and Tissues

#### 2.9.1. Standards Solutions

The two compounds were individually dissolved in MeOH at a concentration of 1 mg/mL (stock solutions) and stored at −20 °C until use. They were mixed in MeOH/water (50/50, *v/v*) to 100 µg/mL for each. Appropriate dilutions were prepared to establish the following range points: 1, 2.5, 5, 10, 25, 50, 100, 250, and 500 ng/mL for ε-viniferin and 25, 50, 100, 250, 500, 1000, 2500, and 5000 ng/mL for resveratrol. 

#### 2.9.2. Stilbenes Extraction from Plasma and Liver Samples

Stilbenes extractions of plasma and liver (one sample of each per rat) were performed as previously described with some modifications [34]. Plasmas (360 µL) were mixed with 1080 µL of cold MeOH and vortexed during 3 min. Samples were centrifuged during 30 min at 12,000 *g* and 4 °C. Supernatants were completely evaporated using SpeedVac concentrator equipped with refrigerated vapor trap (Thermo Fisher Scientific, Waltham, MA USA). Livers (about 0.5 g) were crushed with 3.5 mL MeOH/water (80/20, *v/v*) using Ultra-Turrax homogenizer (IKA, Staufen, Germany) at 12,000 rpm. After centrifugation (10,000 *g*, 20 min, 4 °C), 3 mL of supernatants (A) were stored. Pellets were extracted with 1 mL cold MeOH, vortex-mixed and exposed to ultrasonic bath (1 min). Supernatants (B) obtained after centrifugation (10,000 *g*, 20 min, 4 °C) were pooled with supernatants (A) and evaporated to dryness using SpeedVac concentrator equipped with refrigerated vapor trap. 

Dry plasma and liver were stored at −80 °C before being reconstituted in MeOH/water (50/50, *v/v*), vortex-mixed, exposed to ultrasonic bath and centrifuged (12,000 *g*, 30 min, 4 °C). Supernatants were collected and filtered through PTFE filters before injection of 5 µL into UPLC- Heated Electrospray Ionization (HESI)-HRMS system.

#### 2.9.3. Extracts Analysis by UPLC-HESI-HRMS 

UPLC-HESI-HRMS was constituted by a Vanquish UPLC system coupled with a Q Exactive Plus Hybrid Quadrupole-Orbitrap Mass Spectrometer (Thermo Fisher Scientific, Waltham, MA USA). C18 column was used for the chromatographic separation (Zorbax SB-C18 100 mm × 2.1 mm i.d., 1.8 µm column, and 5 mm × 2.1 mm i.d., guard column, Agilent). Samples were eluted with Milli-Q water (solvent A) and acetonitrile (solvent B) acidified with formic acid (0.1%, *v/v*) at 0.4 mL/min using the following gradient: 0 min (10% B), 1.7 min (10%B), 3.4 min (20%B), 5.1 min (30% B), 7.8 min (30% B), 8.5 min (35% B), 10 min (45%B), 10.5 (100%B), 12 min (100%B), 12.2 min (10%B), 14 min (10%B). The column was maintained at 30 °C and the autosampler at 10 °C. 

Source parameters have been configured in negative mode as follows: HESI II probe heater temperature and capillary temperature were maintained at 350 °C and 370 °C, respectively. Sheath gas (nitrogen) flow rate was set to 35 (a.u), auxiliary gas to 20 (a.u), and sweep gas flow rate to 2 (a.u). The S-Lens RF level was 60 and the spray voltage was 3.5 kV. Targeted-SIM-ddMS2 method was used with the following parameters: SIM was configured with resolution, AGC target and isolation window of 35,000, 1e5, and 1.0 m/z, respectively. A resolution of 17,500 and normalized collision energy of 35 were used for dd-MS^2^. Each peak was integrated manually, and the elemental composition of ions was confirmed using m/z (delta ppm < 5) after extracting the suitable filter spectrum (m/z (226.5714–227.5714), (402.6035–403.60350, (452.6344–453.6344), and (628.6664–629.6664), for resveratrol, resveratrol-glucuronide, ε-viniferin, and ε-viniferin-glucuronide, respectively). Specificity of each peak was verified by comparing chromatograms of samples to those of the blank matrix (control animals). Total resveratrol-glucuronides and total ε-viniferin-glucuronides were expressed as equivalent of their native form. 

### 2.10. Histopathogical Analysis

The histopathological examination of stomach and liver of control and exposed rats was observed with light microscopy (LM). Formaldehyde (10%) at 4 °C was used to fix the samples, and then they were dehydrated with ethanol, immersed in xylol and embedded in paraffin wax. Tissue sections of 3–5 mm were deparaffinized followed by rehydration, stained with hematoxylin and eosin (HE), and mounted with Crystal/Mount (Paraplast, Oxford Labware, St. Louis, MO, USA). 

### 2.11. Statistical Analyses

MN test results are presented as mean ± SD, and the analysis of variance (One-way ANOVA) was performed followed by Tukey multiple comparison test. For the parameters PCE/NCE, PCE/total, and standard and enzyme modified comet assays, mean ± SD of the medians were calculated for each group. The distribution of the data was checked for normality using the D’Agostino–Pearson test and the different groups were compared using the non-parametric Kruskal–Wallis test followed by Dunn’s multiple comparison test. The analyses were performed using Graph-Pad Prisma 9 version 9.0.0 software.

## 3. Results

### 3.1. Micronucleus Test 

No significant difference in the PCE/NCE and PCE/total erythrocytes ratio between the groups treated with ST-99 extract and the negative control group was observed in either sex. In addition, the extract did not increase the % MN in immature erythrocytes at any dose tested (90, 180, and 360 mg ST-99/kg b.w.) compared to control group. In contrast, treatment with the positive control (EMS) induced significant decreases in PCE/NCE and PCE/total and significant increases in %MN-PCEs versus to the respective control group (Table 1).

### 3.2. Standard and Enzyme-Modified Comet Assay

**Figure 1:**
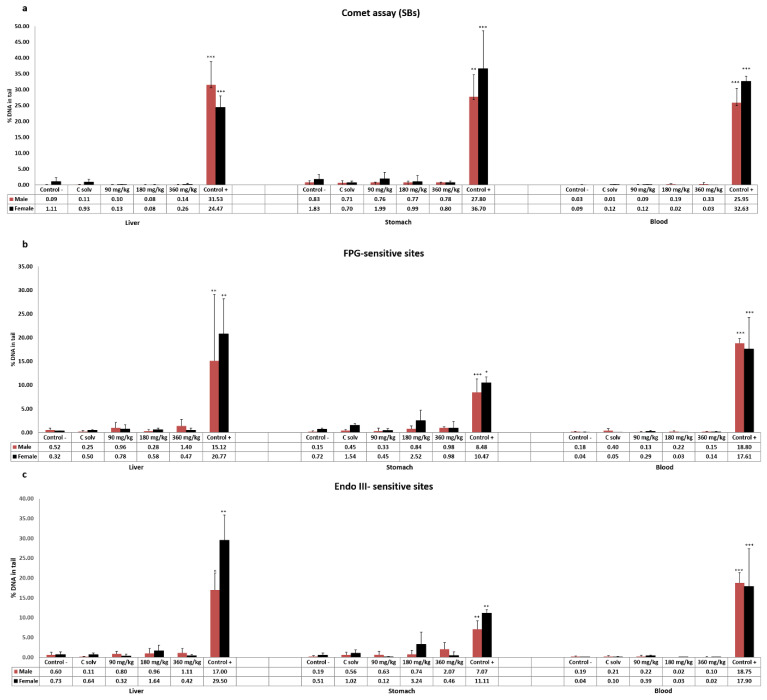
The level of DNA damage measured on cells isolated from liver, stomach, and blood of male and female rats exposed to ST-99 as the formation of strand breaks (SBs) by the standard comet assay (**a**), and oxidative DNA damage as Endo III-sensitive sites (**b**) and FPG-sensitive sites (**c**) by the modified comet assay. The levels of DNA strand breaks and oxidized pyrimidines/purines are expressed as % DNA in tail. All values are expressed as mean ± SD. The significant levels observed are * *p* < 0.05, ** *p* < 0.01, or *** *p*< 0.001.

The results obtained in the standard comet assay after exposure of Wistar rats to ST-99 extract are showed in Figure 1a. No DNA strand breaks were induced at any assessed dose in liver, stomach, and blood cells in both male and female rats.

Moreover, the enzyme-modified comet assay was performed to determinate oxidative DNA damage. The results indicated that the exposure of 90, 180, and 360 mg ST-99/kg b.w. in both sexes did not induce an increase in the frequency of Endo III or FPG-sensitive sites in any tissue assayed compared to the control group (Figure 1b,c).

For both assays, significantly different response (*p* < 0.05, *p* < 0.01, or *p*< 0.001) of the positive control groups treated with 200 mg/kg b.w. of EMS with respect to the control groups was found.

### 3.3. Presence of ε-Viniferin, Trans-Resveratrol, and Its Derived Compounds in Tissue

**Figure 2:**
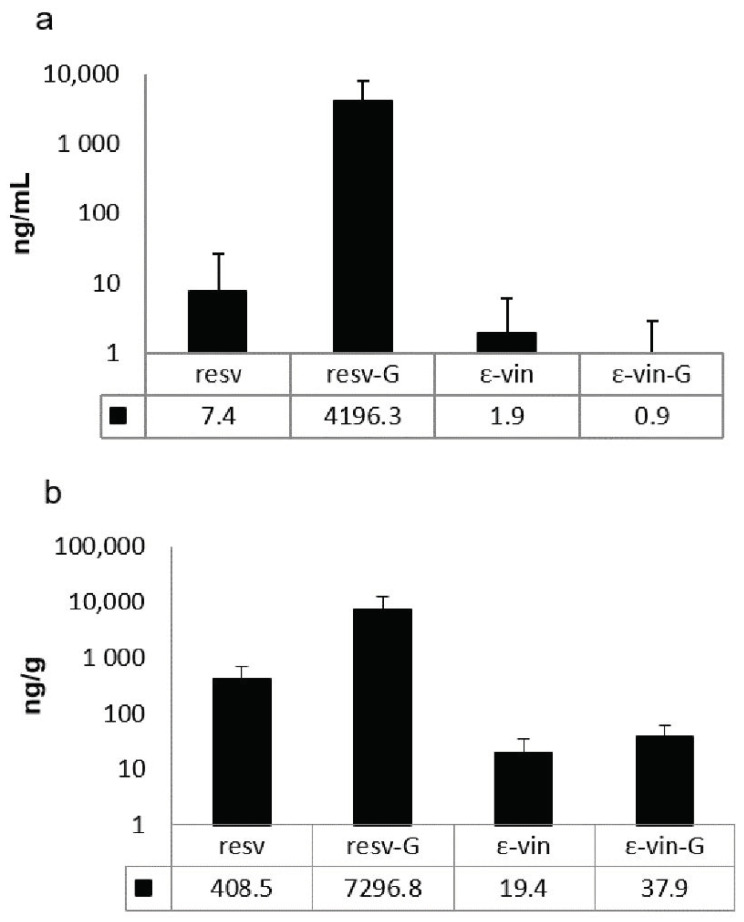
(**a**) Plasma and (**b**) liver concentrations of resveratrol (resv), total resveratrol-glucuronides (resv-G), ε-viniferin (ε-vin), and total ε-viniferin-glucuronide (ε-vin-G) 3 h after final dose of 360 mg ST-99/kg b.w Data are expressed as mean ± SD in ng/mL and ng/g for plasma and liver, respectively (*n* = 10).

The results indicated that derivatives of both ε-viniferin and trans-resveratrol as well as the native forms are found in plasma and liver of the rats treated at the highest concentration tested 3 h after the final administration of ST-99 (Figure 2). In the plasma, the main compounds were resveratrol-glucuronides which represent more than 99% of total detected compounds (Figure 2a). In liver, the glucuronide metabolites of resveratrol are also the major compounds but to a lesser proportion (>90%) (Figure 2b). Unmetabolized and metabolized ε-viniferin were present in plasma and liver. The compounds were present in very low concentrations compared to those of resveratrol.

### 3.4. Clinical and Histopathological Analysis

**Figure 3:**
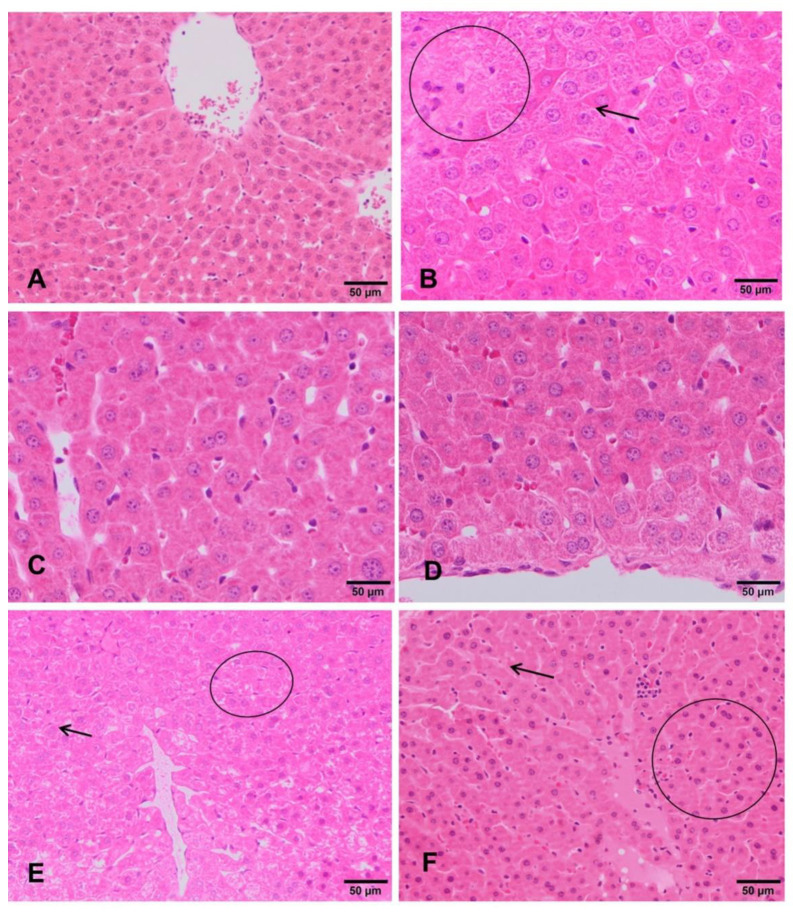
Histopathological changes in the liver of rats exposed to ST-99. Normal hepatic parenchyma is observed in negative control group (**A**). Detail of hepatic cordons of rat exposed to the positive control (**B**), showing glycogenic degeneration (arrow) and abundant polyploid hepatocytes (circle). Rats exposed to 90 and 180mg/kg ST-99 showed an apparently normal liver parenchyma (**C**,**D**). Rats exposed to 360 mg/kg ST-99 exhibited slight presence hepatic apoptosis throughout the lobule (circle) and glycogenic degeneration (arrow) (**E**,**F**).

The livers of negative control rats showed an unaltered liver parenchyma (Figure 3A). Hepatocytes arranged in cords can be observed, with a radial distribution to the centrilobular vein. These cells maintain their polyhedral morphology and bipolarity, with a spherical nucleus standing out in the center of the cell showing an evident nucleolus. The cytoplasm is homogeneous and acidophilic. Similar appearance was observed in both sexes. Rats exposed to the lowest doses (90 mg/kg b.w and 180 mg/kg b.w.) showed that the architecture of the lobule is apparently normal with respect to the hepatic cords, the hepatocytes, the sinusoid capillaries, and its orientation to the centrilobular veins (Figure 3C,D). However, in rats treated with 360 mg/kg b.w., glycogenic degeneration processes are observed. Clear cells with small nuclei and basophils showed features evidencing incipient hepatitis (Figure 3E,F). Regarding the positive control group, the hepatocytes still present a radial arrangement in the liver lobule, but they increased in size due to the accumulation of glycogen. The nuclei are mostly morphologically normal, they are ovoid with intense basophilia and smaller than the negative control group. The rest of the cytoplasm is very clear. Cells are undergoing an apoptosis death. All these morphological features correspond to hepatitis with a process of glycogenic and necrotic degeneration (Figure 3B).

**Figure 4:**
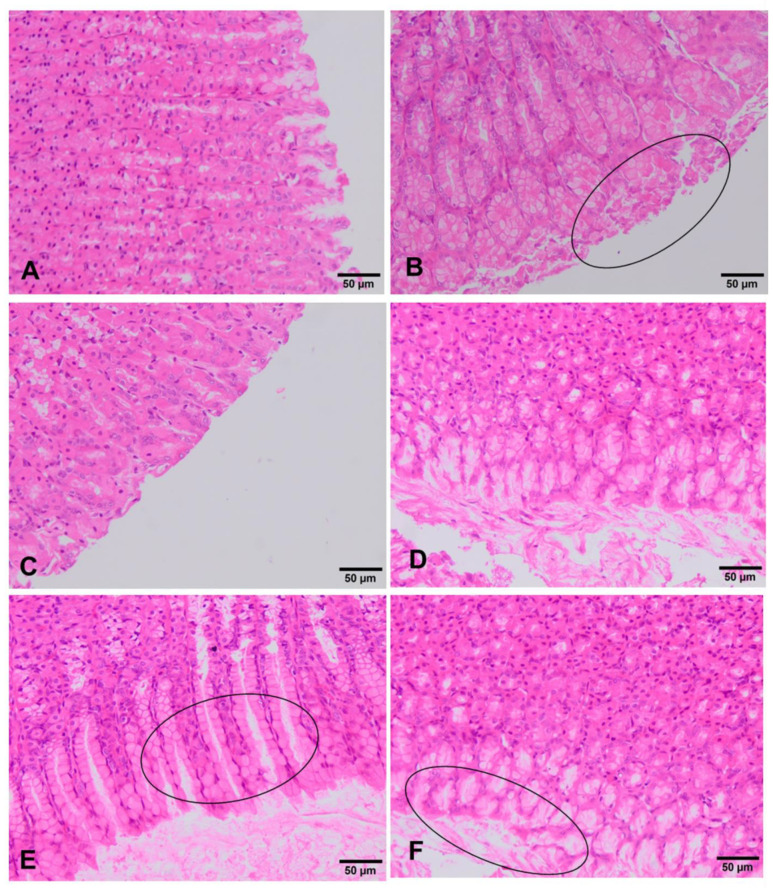
Histopathological changes in the stomach of rats exposed to ST-99. Apparently normal gastric mucosa is observed in negative control group (**A**). In positive control rats (**B**) gastric mucosa with processes of desquamative necrotic gastritis (circle) are observed. Rats exposed to 90 mg/kg ST-99 showed an apparently normal gastric mucosa (**C**). Unaltered gastric mucosa are observed in rats treated with 180 mg/kg ST-99 (**D**). Rats exposed to 360 mg/kg ST-99 exhibited desquamative catarrhal gastritis (circle) (**E**,**F**).

In the stomach of unexposed rats, no type of alteration was observed in the different cells of the mucosa. There are no differences between both sexes in all the experimental groups. Rats exposed to the lowest doses (90 mg/kg b.w. and 180 mg/kg b.w.) showed no remarkable change, showing in both sexes a similar histology to that described above for the negative control group (Figure 4A,C,D). However, in the group treated with 360 mg/kg, a film of mucus is observed on the external part of the gastric mucosa. Considering that necrosis does not occur, this finding may correspond to a process of slight scaly catarrhal gastritis (Figure 4E, F). The positive control group shows in both sexes a gastric mucosa with necrotic and desquamated cells and lined by a mucous membrane that includes remains of mucous cells (Figure 4B).

## 4. Discussion

The genotoxic evaluation must be addressed as part of the evaluation process of any new additive since genetic alterations in somatic and germ cells could lead to serious health effects [23]. In this sense, in vitro genotoxic studies were performed with ST-99 and revealed contradictory results. Exposure to this extract showed negative results in the Ames test and in the standard and enzyme-modified comet assay. However, genotoxic effects were obtained in the MN test in presence of the S9 mix [19]. Therefore, in order to ensure whether the genotoxic response in vitro was expressed in vivo, an appropriated in vivo study is mandatory. The in vivo genotoxicity of ST-99 has not been studied yet, and similarly, no extract containing stilbenes has been fully studied in this sense. Up to date, there is only one report which evaluated the capacity of melinjo (Gnetum *gnemon* L.) seed extract, rich in *trans*-resveratrol and dimers and glycosides of resveratrol, by an MN test in rats by gavage for 2 days (1000, 2000, or 4000 mg/kg b.w./day). This extract did not increase the incidence of the number of micronucleated immature erythrocytes. These results suggested that melinjo seed extract does not have genotoxic potential to induce chromosome aberrations in mammals [22]. Similarly, in our work, no increase in the number of micronucleated cells in any of the treated groups of both sexes was observed. On the contrary, when L5178Y Tk^+/−^ cells were exposed to ST-99, a significant increase in binucleated cells was observed with metabolic activation at doses up to 60 µg/mL [19]. Kirkland et al. [35] indicated that these differences may be because of the deficiencies in metabolism, p53 function, and DNA repair capability of most of the rodent cell lines used. The FDA Toxicological Principles for the Safety Assessment of Food Ingredients [36], stated that positive genotoxicity results that may not be relevant in vivo, may arise in vitro due to changes in pH, osmolality or high levels of cytotoxicity. Moreover, the substances are usually less toxic in in vivo models since detoxification processes may occurred [18,35]. In this sense, the in vivo MN test has been preferably recommended in comparison to the in vitro MN [37]. Hence, based on the weight of evidence presented above, ST-99 extract is unlikely to exhibit genotoxicity. 

Negative genotoxicity results were also obtained when resveratrol was studied individually in vivo experimental models. Hynes [38] performed MN test using Sprague–Dawley rats given 0, 500, 1000, or 2000 mg/kg b.w./day *trans*-resveratrol for 48 h consecutive by gavage. The absence of clastogenic activity in vivo *of trans*-resveratrol was demonstrated since no increase in micronucleated erythrocytes was observed at any dose of resveratrol [38,39]. Moreover, Resvida^TM^, a pure *trans*-resveratrol preparation, was evaluated for potential induction of MN micronucleus in Sprague–Dawley rat bone marrow cells following OECD test Guideline 474. Resvida ^TM^ was non-genotoxic at up 2000 mg/kg b.w./day [40]. To our knowledge, no in vivo genotoxicity studies evaluating the safety of *trans*-ε-viniferin have been described in the scientific literature.

The comet assay has become widely used to measure the DNA damage and the detection of altered bases. Moreover, the range of DNA lesions that can be detected has been increased by the use of repair enzymes [41]. The incorporation of DNA digestion Endo III and FPG, allows the measurement of oxidized pyrimidines and oxidised purines, respectively [21,42]. Our results of alkaline comet assay showed that the ST-99 extract exposure did not produce DNA breaks in cells isolated from either the stomach or the liver cells of rats. This agrees with the in vitro negative results previously reported [19]. Similarly, Attia [43] showed that 7 days of oral administration of resveratrol (100 mg/kg b.w.) did not induce any increase in DNA strand breaks in the mouse bone narrow. Moreover, no DNA damage in the liver and kidney tissues of Wistar rats was observed at a dose of 100 mg/kg b.w. intra-peritoneal [44].

The application of the enzyme-modified comet assay was interesting since depending on the reaction conditions, their concentration, time of exposure, and cell type, it is not uncommon for phytochemicals compounds to show both antioxidant and prooxidant activities. This could give place to increasing amounts of oxidizing free radicals, oxidative breakage of cellular DNA, protein and lipid damage, and thereby modulate/trigger initiation, promotion, and progression of cancer [45,46,47,48,49]. In this work, no significant changes were observed in the % DNA in tail in Endo III or FPG-sensitive sites in both stomach and liver cells. This is in agreement with previous work that reported that no significant changes were observed after 24 h or 48 h exposure to ST-99 extract at 4.82–27.79 µg/mL and at 6.64–31.92 µg/mL in Caco-2 cells and Hep-G2, respectively analyzed with FPG post-exposure [19]. These researchers reported that ST-99 extract at low concentrations reduced reactive oxygen species (ROS) and exhibited DNA-protective effects against oxidizing agents such as H_2_O_2_ and Ro19-8022 in colon and hepatic cell lines (Caco-2 and HepG2 cells) [16,19]. Focusing on studies evaluating the effects of resveratrol on oxidative DNA damage, there were no statistically significant differences in the FPG-modified comet assay in the liver and kidney cells of rats between the control and the resveratrol-treated groups [44].

Following the recommendations of the OECD 474 and 489 [25,26], because of the absence of genotoxicity in both assays and the no detection of a decrease in PCE/NCE ratio in the exposure groups, it is necessary to demonstrate the presence of stilbenes in the target tissues. In this sense, evidence of exposure of the bone marrow to a substance can be determined if there is a decrease in the ratio between immature and mature erythrocytes. However, in our assay, this parameter did not change between the control and the treated rats, and therefore, following the protocol recommendations, we carried out an analytical study in order to confirm the presence of stilbenes in blood using high pressure liquid chromatography coupled to a mass spectrometer (UPLC-HESI-MS). This study is of great interest since the bioavailability of stilbenes is low. *Trans*-resveratrol has a bioavailability that ranges between 29% and 38% due to several factors: low solubility in water, short half-life, and rapid metabolism [50,51,52,53]. Likewise, the bioavailability of *trans*-ε-viniferin has been reported in mice, being extremely low (0.77%) [34,54]. However, when we evaluate the bioavailability of a botanical mixture, we must consider that there may be interactions between its components affecting this parameter [55].

The UPLC-HESI-MS analysis of plasma and liver indicated the presence of compounds derived from the ST-99. Glucuronic forms are the main metabolites found, but resveratrol-sulphated forms were also detected but non-quantifiable (data not shown). The low concentrations found of native molecules (resveratrol and ε-viniferin) are in accordance with bioavailability studies after oral administrations and explained by low absorption and an intense hepatic metabolism [34,54,56,57,58,59]. The presence of these stilbenes and their metabolites is of great interest since the exposure of the target organs of this study is confirmed.

In relation to histopathological studies, the ST-99 extract at the lowest doses tested (90 mg/kg b.w. and 180 mg/kg b.w.) did not induce relevant histopathological damage in liver and stomach tissues. However, at 360 mg/kg b.w. of the extract, slight damage was detected in both organs. Incipient hepatitis and desquamative catarrhal gastritis were observed. By contrast, severe morphological changes were detected in in vitro studies when the Hep-G2 cells were exposed to three different concentrations of the extract and the mixture of stilbenes (*trans*-resveratrol and *trans*-ε-viniferin). The hepatic human cell line HepG2 exposed to 31.91 µg/mL of the extract showed cytoplasmatic projections that would turn into apoptotic bodies. Moreover, ST-99 induced breakdown in the cell cycle by inhibiting cell proliferation and caused cell death mainly by apoptosis. This effect was minimized with the treatment with the mixture of stilbenes [17]. Moreover, the concentrations tested in this work are higher than the used in in vitro assays, then, the lack of effects detected in vivo may be because the differences in metabolism and the bioavailability of the extract to the target organ or because different species were used, rats in in vivo study and human cell line in vitro.

Overall, the results obtained in the combined MN and comet assay carried out in rats indicate that the ST-99 extract has no genotoxic potential at the concentrations tested (90, 180, and 360 mg/kg of b.w.). In addition, the presence of stilbenes and their metabolites were found in plasma and tissues evidencing the exposure of these tissues to this extract. Moreover, although a slight histopathological damage has been showed in the stomach and liver, it was only at the highest concentration tested, which is 100 times higher than the amount that would reach humans in a regular consumption of wine containing the extract as an additive. Therefore, the present work confirms that ST-99 is not genotoxic. These findings are of great interest since wines treated with natural preservative will be more competitive in the current global market. More studies are required to determinate the effective and safe doses to be used in the industry. The possible effects of plant extracts on quality and sensory properties of wines need also to be assessed. In addition, in wine producing zones, the biological origin of the extract presents an important advance in environmental protection thanks to the revaluation of by-products and the reduction of forestry wastes.

## Figures and Tables

**Table 1:** Micronucleus assay results in male (♂) and female (♀) rats. Bone marrow cytotoxicity expressed as polychromatic erythrocytes (PCE) among total erythrocytes (normochromatic erythrocytes (NCE) + PCE), ratio PCE among NCE, and the micronuclei induction expressed as % micronucleus (MN).

Groups	Sex	*n*	Doses	PCE/NCE	PCE/Total	% MN
Negative Control	♂	5		1.15 ± 0.17	0.53 ± 0.04	0.87 ± 0.08
	♀	5		1.78 ± 0.10	0.64 ± 0.03	0.99 ± 0.08
Solvent Control	♂	5		1.09 ± 0.19	0.51 ± 0.04	0.91 ± 0.16
	♀	5		2.35 ± 0.73	0.69 ± 0.07	1.03 ± 0.07
Positive Control	♂	3	200 mg/kg	0.50 ± 0.08 **	0.33 ± 0.03 *	2.35 ± 0.06 ***
	♀	3		0.68 ± 0.36 **	0.39 ± 0.13 *	2.39 ± 0.05 ***
ST-99 extract	♂	5	90 mg/kg b.w.	0.94 ± 0.49	0.46 ± 0.12	0.86 ± 0.12
	♀	5		1.12 ± 0.39	0.51 ± 0.08	0.89 ± 0.14
	♂	5	180 mg/kg b.w.	0.90 ± 0.17	0.46 ± 0.04	0.85 ± 0.05
	♀	5		1.16 ± 0.57	0.51 ± 0.10	0.93 ± 0.09
	♂	5	360 mg/kg b.w.	0.84 ± 0.38	0.43 ± 0.12	0.89 ± 0.19
	♀	5		1.32 ± 0.22	0.56 ± 0.04	0.94 ± 0.17

All values are expressed as mean ± SD. The significant levels observed are * *p* < 0.05, ** *p* < 0.01, or *** *p* < 0.001.
